# Dental Works in the Enoch Pratt Free Library

**Published:** 1894-11

**Authors:** 


DENTAL WORKS IN THE ENOCH PRATT FREE LIBRARY.—Recently we noted the fact that Dr. Bernard C. Steiner, the librarian, had expresed a willingness to add to the books on dentistry in the Pratt Library, which were four in number, namely, Arthur's "Prevention of Decay of the Teeth," Meredith's "The Teeth and How to Save Them," Shaw's "Treatise on Odontalgia", and "Letters from a mother to a mother on the formation, growth and care of childrens teeth."

The new volumes which are standard, and will be of use to the practicing dentists who may not have these in their private libraries are:

Evans—Crown and Bridge Work.

Black—Anatomy of the Human Teeth.

Richardson—Mechanical Dentistry.

Webb—Notes on Operative Dentistry.

Garretson—Oral Surgery.

Harris—Dictionary of Dental Science (Revised Edition by Dr. F. J. S. Gorgas.)

Kingsley—Oral Deformities.

Talbott—Irregularities.

Essig—Dental Metallurgy.

Taft—Index to Dental Literature.

Haskell—Students' Manual and Handbook for the Dental Laboratory.

Guilford—Orthodontia. R. G.

